# Role of lectin pathway complement proteins and genetic variants in organ damage and disease severity of systemic sclerosis: a cross-sectional study

**DOI:** 10.1186/s13075-019-1859-1

**Published:** 2019-03-18

**Authors:** Michael Osthoff, Veronika K. Jaeger, Ingmar A. F. M. Heijnen, Marten Trendelenburg, Suzana Jordan, Oliver Distler, Ulrich A. Walker

**Affiliations:** 1 Department of Internal Medicine, University Hospital Basel, 4031 Basel, Switzerland; 2 Department of Biomedicine, University Basel, 4031 Basel, Switzerland; 3 Department of Rheumatology, University Hospital Basel, 4031 Basel, Switzerland; 4 Division of Medical Immunology, Laboratory Medicine, University Hospital Basel, 4031 Basel, Switzerland; 5 Department of Rheumatology, University Hospital Zurich, 8091 Zurich, Switzerland

**Keywords:** Mannose-binding lectin, Ficolin-2, Systemic sclerosis, Complement system, Innate immunity

## Abstract

**Background:**

The role of the complement system in the pathogenesis of systemic sclerosis (SSc) is controversial. This study investigated the role of the lectin pathway of complement as a mediator of ischemia/reperfusion injury in SSc.

**Methods:**

This is a prospective observational cross-sectional study of 211 SSc patients and 29 patients with Raynaud’s phenomenon in undifferentiated connective tissue disease (UCTD) at risk of developing SSc from two outpatient clinics. Serum levels of lectin pathway proteins (FCN-2, FCN-3, MBL, and MASP-2) and eight *MBL2* and *FCN2* single-nucleotide polymorphisms (SNP) were analyzed by sandwich-type immunoassays and genotyping and examined for their association with disease manifestations.

**Results:**

Lectin pathway protein levels and SNPs were similar between SSc and UCTD patients. FCN-2 levels were however higher in SSc patients with present evidence of digital ulcers (mean 1.4 vs. 1.0 μg/mL, *p* = 0.05), pitting scars (mean 1.3 vs. 1.0 μg/mL, *p* = 0.01), and puffy fingers (mean 1.2 vs. 1.0 μg/mL, *p* = 0.04). Similarly, higher FCN-2 levels were observed in SSc patients with Scl-70 autoantibodies (mean 1.5 vs. 1.0 μg/mL, *p* = 0.001), interstitial lung disease (mean 1.2 vs. 0.9 μg/mL, *p* = 0.02), and a forced vital capacity (FVC) below 80% (mean 1.4 vs. 1.0 μg/mL, p = 0.02). In line, variant alleles in the *FCN-2* SNP at position + 6359 were associated with a significantly reduced FVC and diffusion capacity. Furthermore, patients with SSc renal crisis harbored higher MBL levels (mean 2.7 vs. 1.5 μg/mL, *p* = 0.04). No other lectin pathway protein levels or polymorphisms were associated with disease manifestations, low complement C3 and/or C4 levels, or inflammatory markers.

**Conclusions:**

This study does not support a relevant role for several lectin pathway complement proteins in the pathogenesis of SSc. Higher FCN-2 levels were however associated with Scl-70 autoantibody positivity, interstitial lung involvement, and digital vasculopathy. Elevated MBL levels were associated with renal crisis.

## Background

Systemic sclerosis (SSc) is a difficult-to-treat orphan disease that is associated with a considerable morbidity and mortality [1]. The pathophysiological processes that underlie SSc are complex and not completely understood. Vascular injury with a subsequent dysregulation of tissue repair has been proposed as an important paradigm [2, 3]. Dysfunctional innate and adaptive immune responses and microvascular injury contribute to the clinical phenotype. Endothelial cell damage and microvascular dysfunction are early and important events in the pathogenesis of SSc and drive the activation of the immune system [4, 5]. The complement system, a complex cascade of circulating and surface-bound proteins that play an essential role in the innate immune defense, has been implicated in certain rheumatic diseases, such as rheumatoid arthritis and systemic lupus erythematosus [6, 7], but its role in SSc is still debated. Abnormal local complement activation has been detected in skin biopsies of SSc patients [8], and low serum complement levels were also part of the first European Scleroderma Trials and Research group activity index [9]. However, in several subsequent studies, hypocomplementemia was not associated with SSc disease activity [10–12].

In the presence of endothelial cell damage, the lectin activation pathway of complement has a central role in ischemia/reperfusion (IR) injury. After binding of the pattern recognition receptors (PRR) mannose-binding lectin (MBL), collectin liver 1, and ficolins (FCN-1, FCN-2, and FCN-3) to their target structures on pathogens or stressed and dying cells, MBL-associated serine proteases (MASP)-1 and MASP-2 are activated. In addition to downstream complement activation, MASP-1 cleaves substrates of the kinin and coagulation system and activates endothelial cells, thus eliciting clot formation and conveying proinflammatory signaling (reviewed in [13]). Several lines of evidence suggest that the lectin pathway aggravates IR injury not only in experimental models but also in humans [14–16].

Removal of cell debris by PRR has also been implicated in the pathogenesis of several autoimmune diseases [17–19]. Three studies have recently associated MBL, FCN-2, and FCN-3 serum concentrations with disease severity and organ involvement in SSc [20–22]. The number of patients studied was however small, and the results discordant to some extent. Given the central role of the lectin pathway in IR injury and its interaction with stressed endothelial cells and the coagulation system, we aimed to comprehensively assess the association of the lectin pathway of complement with organ involvement and disease severity in a larger group of SSc patients. We also included a group of patients with Raynaud’s phenomenon in undifferentiated connective tissue disease (UCTD) at risk of developing SSc to study an early time point in the course of the disease [23, 24].

## Methods

### Participants

We conducted a cross-sectional prospective study of Raynaud’s phenomenon in undifferentiated connective tissue disease (UCTD) at risk of developing SSc [23, 24] and SSc patients enrolled at the University Hospitals of Basel and Zurich as part of the European Scleroderma Trials and Research group centers (EUSTAR) [25]. Following approval by the Human Research and Ethics Committee at participating Basel and Zurich University SSc centers, centers were required to provide informed written consent. Since lectin pathway proteins had previously been assessed in a healthy control group compared to SSc patients [18, 19] and because of our primary aim to investigate organ involvement and disease severity in SSc patients, a control group of healthy subjects was not included in the present study.

For inclusion into the study, SSc patients were required to fulfill the 1980 American College of Rheumatology (ACR) [26] or the 2013 ACR/EULAR classification criteria [27] and to be over 18 years of age. Patients were classified into limited or diffuse SSc depending on the most severe skin involvement during follow-up. UCTD patients were required to suffer from Raynaud’s phenomenon (RP) in addition to other features compatible with early SSc such as the presence of puffy fingers, antinuclear antibodies, or an abnormal nail fold capillaroscopy, but not to fulfill the 2013 ACR/EULAR classification criteria for SSc [24, 27]. Clinical data at the most recent visit were used for the purpose of this study. The presence or absence of pulmonary hypertension (PH) was recorded according to the judgment of the SSc expert in the center including an estimation of the systolic pulmonary arterial pressure > 40 mmHg on transthoracic echocardiography, or confirmation by right heart catheterization. Interstitial lung disease (ILD) was defined as the presence of characteristic abnormalities on high-resolution chest computer tomography or X-ray, and scleroderma renal crisis was defined as the presence of at least two of the following: new-onset hypertension, microangiopathic anemia, and rising creatinine. Digital ulcers were defined as current ulcers distal to or at the proximal interphalangeal joint. Pitting scars were defined as scars distal to or at the PIP joint not thought to be due to trauma. Disease duration was calculated from the onset of the first non-Raynaud’s phenomenon SSc symptom.

### Determination of lectin protein serum concentration and genetic variants

Quantification of MBL serum levels was performed in duplicates by an investigator blinded to clinical data using a mannan-binding enzyme-linked immunosorbent assay (ELISA) as previously described [28]. Briefly, mannan-coated microtiter plates were incubated with samples at 1:25 and 1:100 dilutions for 90 min at room temperature followed by detection of bound MBL with a biotinylated monoclonal anti-MBL antibody (HYB 131-01, BioPorto Diagnostics, Gentofte, Denmark). Subsequently, plates were developed with tetramethylbenzidine substrate solution (BD OptEIA, Becton Dickinson, Switzerland) after incubation with ExtrAvidin peroxidase conjugate (Sigma-Aldrich, Allschwil, Switzerland), stopped with 1 M H_2_SO_4_ (Sigma-Aldrich), and read immediately on a plate reader. MBL concentrations were calculated against a standard pool serum (BioPorto Diagnostics). MBL deficiency was defined as a serum level below 0.5 μg/mL [29]. FCN-2, FCN-3, and MASP-2 serum levels were quantified using commercially available ELISA kits (Hycult Biothech, Uden, The Netherlands).

For genetic analyses, genomic DNA was extracted. Four *MBL2* (rs1800451, rs1800450, rs5030737, and rs7096206) and four *FCN2* (rs3124953, rs17514136, rs17549193, and rs7851696) promoter or exon polymorphisms were determined by allele-specific polymerase chain reactions (PCR) using TaqMan fluorescent probes (TaqMan genotyping assays, Thermo Fisher Scientific, Lausanne, Switzerland) as described elsewhere [30]. Promoter and exon polymorphisms were analyzed separately and combined as haplotypes [31].

### Statistical analysis

We used chi-square tests for comparisons of categorical variables and allele frequencies (dominant model of inheritance, i.e., wild-type vs. hetero- and homozygous) and to check for the Hardy–Weinberg equilibrium. Lectin pathway protein concentrations were analyzed by means of the Mann–Whitney *U* test with regards to differences in disease severity and organ involvement, then reporting median and interquartile ranges (IQR) or by means of the Student’s *t* test (then reporting mean and standard deviation (SD)), as appropriate. Continuous variables were correlated with lectin pathway protein concentrations using Pearson correlation coefficients. Haplotype frequencies were analyzed by means of the expectation-maximum algorithm. Haplotype and linkage disequilibrium analysis was carried out with the Haploview program (version 4.2). All other analyses were performed with Stata/IC 13.1 (StataCorp, College Station, USA).

## Results

### Demographic and clinical characteristics

The study population consisted of 211 patients with established SSc and 29 UCTD patients with visits between 2005 and 2016. Of the SSc patients, 3 patients (1%) only fulfilled the 1980 ACR criteria [26], 38 patients (18%) only fulfilled the 2013 ACR/EULAR criteria [27], and 170 (81%) fulfilled both criteria. The demographics and clinical characteristics are outlined in Table 1.

**Table 1:** Characteristics of SSc patients and patients with VEDOSS

Characteristics	SSc patients (*n* = 211)	UCTD (*n* = 29)	All patients (*n* = 240)
Female, %	79	93	80
Age (years), mean (SD)	60 (13)	51 (14)	59 (14)
Caucasian race, %	97	100	97
Duration since RP onset (years), mean (SD)	14 (12)	8 (10)	13 (12)
Duration since first non-RP (years), mean (SD)	13 (11)	na	na
Diffuse disease, %	18	na	na
Autoantibodies, %			
Anti-topoisomerase I (Scl-70)	29	7	
Anti-centromere	45	34	
Anti-RNA polymerase III	8	3	
Organ involvement,%			
Esophageal symptoms	48	31	45
Pulmonary hypertension	15	0	13
Interstitial lung disease	49	0	42
Renal crisis	2	0	2
Disease activity/severity			
Digital ulcers, %	12	0	10
mRSS, median (IQR)	4 (2–9)	0 (0–0)	3 (0–9)
EUSTAR SSc activity score, median (IQR)	1 (0.5–2.0)	na	na
FVC (% predicted), mean (SD)	98 (21)	99 (15)	98 (20)
DLCO/va (% of predicted), mean (SD)	79 (16)	94 (17)	82 (17)
DLCO/sb (% of predicted), mean (SD)	71 (19)	96 (13)	74 (20)

Abbreviations: *DLCO* diffusing capacity of the lung for carbon monoxide, *EUSTAR* European Scleroderma Trials and Research Group, *FVC* forced vital capacity, *mRSS* modified Rodnan skin score, *RP* Raynaud’s phenomenon, *SD* standard deviation, *SSc* systemic sclerosis, *na* not applicable

Mean age (SD) was 60 (13) and 51 (14) years in SSc and UCTD patients, respectively, and 166 (79%) and 27 (93%) were female. Characteristics of UCTD patients included the presence of antinuclear antibodies in 27 (93%), of puffy fingers in 2 (7%), and of abnormal nailfold capillaroscopy in 16 (55%) of patients. SSc patients were classified as limited and diffuse SSc subsets in 36% and 18%, respectively, with the remaining patients featuring only sclerodactily (26%) or being classified as sine scleroderma (20%). Anti-centromere, anti-Scl-70, and anti-RNA polymerase III antibodies were present in 45%, 29%, and 8% of SSc cases, respectively. ILD was the most frequently encountered organ involvement (49%).

### Lectin pathway proteins in UCTD patients vs. those with established SSc

As expected, the time since Raynaud’s phenomenon (RP) onset was significantly shorter in UCTD compared to SSc patients (mean 8.0 years (SD 10.0) vs. 14.2 years (SD 11.7), *p* = 0.007). Similarly, the prevalence of skin and other organ involvement was lower in UCTD patients (Table 1). *MBL2* and *FCN2* allele frequencies at all eight positions were in agreement with the predicted Hardy–Weinberg equilibrium when including all analyzed patients (data not shown).

Serum concentrations of the lectin pathway proteins MBL, FCN-2, FCN-3, and MASP-2 and frequency of *FCN2* and *MBL2* polymorphisms did not differ between UCTD and SSc groups. Although, MBL deficiency defined as a serum concentration below 0.5 μg/mL was numerically more common in UCTD patients (48.3% vs. 31.3%, *p* = 0.07), MBL serum concentrations (mean 1.1 vs. 1.5 μg/mL, *p* = 0.1) and the frequency of *MBL2* polymorphisms were similar (data not shown). There was no correlation of lectin pathway proteins with the time since RP onset as well as with the speed of disease onset, i.e., the duration between the first non-RP manifestation of the disease and the onset of RP (data not shown).

### Association of lectin pathway proteins with organ involvement and autoantibodies in patients with established SSc

Regarding SSc subsets, lectin pathway protein concentrations and the frequency of *FCN2* and *MBL2* polymorphisms were similar. FCN-2 concentrations were significantly higher in SSc patients with present evidence of digital ulcers, with pitting, and with puffy fingers (Table 2), but not with the presence of sclerodactily, telangiectasia, or joint synovitis, whereas there was no association with other lectin pathway proteins or polymorphisms.

**Table 2:** Association of ficolin-2 concentrations with SSc skin manifestations and organ involvement

Variables	Present (μg FCN-2/ml), mean (SD)	Absent (μg FCN-2/ml), mean (SD)	*p* value
Skin manifestations			
Digital ulcers	1.4 (1.0)	1.0 (0.7)	0.05
Pitting scars	1.3 (0.9)	1.0 (0.7)	0.01
Puffy fingers	1.2 (1.0)	1.0 (0.6)	0.04
Sclerodactily	1.1 (0.8)	1.1 (0.8)	0.9
Telangiectasia	1.1 (0.9)	1.0 (0.7)	0.7
Organ involvement			
PH	0.9 (1.2)	1.0 (0.7)	0.6
ILD by radiography	1.2 (1.1)	0.9 (0.6)	0.02
FVC < 80% predicted	1.4 (1.3)	1.0 (0.7)	0.02
Renal crisis	1.4 (1.1)	1.1 (0.9)	0.5

Abbreviations: *FVC* forced vital capacity, *ILD* interstitial lung disease, *PH* pulmonary hypertension, *SD* standard deviation

In general, the serum concentrations of lectin pathway proteins and the presence or absence of genetic polymorphisms were not associated with the modified Rodnan skin score, the EUSTAR SSc activity score [9, 32], inflammatory markers (ESR), the presence of hypocomplementemia, or disease duration (data not shown). Similarly, associations of lectin pathway protein concentrations or polymorphisms with SSc organ involvement (including pulmonary hypertension, radiographic interstitial lung disease, and pericardial effusion) and its surrogate measurements (including esophageal symptoms, pulmonary artery pressure, diffusion capacity, FVC, left ventricular ejection fraction, and proteinuria), or the presence of antinuclear autoantibodies (ANA) in general and the presence of SSc-specific autoantibodies in particular were absent (data not shown). There were however three important exceptions (Table 2).

First, there were higher FCN-2 concentrations in SSc patients harboring Scl-70 autoantibodies than in patients who were Scl-70 autoantibody negative (mean 1.5 μg/mL (SD 1.2) vs. 1.0 (SD 0.7), *p* = 0.001).

Second, FCN-2 concentrations were associated with a radiographic diagnosis of ILD (Table 2). Additionally FCN-2 concentrations were lower in patients with a FVC below 80% predicted than in those with a FVC above 80% predicted (Table 2). In line with this observation, FVC and diffusing capacity (DLCO) were significantly lower in patients with hetero- or homozygous variant alleles in the *FCN-2* polymorphism at position + 6359 C>T (rs17549193), which are associated with elevated FCN-2 serum concentrations [33], compared to those with the presence of the wild-type allele (mean FVC 95% of predicted (SD 19%) vs. 101% of predicted (SD 21%), *p* = 0.03; mean DLCO 67% of predicted (SD 20%) vs. 74% of predicted (SD 18%), *p* = 0.02).

Third, MBL concentrations were substantially higher in five patients with scleroderma renal crisis (mean 2.7 (SD 2.1) vs. 1.5 (SD 1.3) μg/mL, *p* = 0.04). In line, all five patients with renal crisis were homozygous for the wild-type allele of the *MBL2* exon1 polymorphism at position + 54 G>A (rs18004500) and at position + 52 C>T (rs5030737), which is strongly associated with a high MBL concentration.

## Discussion

The present study examined the association of lectin pathway complement proteins with disease activity and organ manifestations in a large group of SSc patients and for the first time in a small number of patients with Raynaud’s phenomenon in UCTD at risk of developing SSc [23]. Contrary to our a priori hypothesis, we failed to identify any relevant association of several lectin pathway protein levels and *FCN2* or *MBL2* polymorphisms with SSc disease activity or organ involvement. There was however an exception of potential importance, namely an association of higher FCN-2 concentrations with the presence of Scl-70 antibodies, several hallmarks of SSc vasculopathy (digital ulcers, pitting scars and puffy fingers), and fibrotic features (compromise in FVC and radiographic evidence of ILD). In addition, the presence of variant alleles in the *FCN-2* polymorphism at position + 6359 C>T (rs17549193) was associated with a decreased FVC and a diminished diffusion capacity compared to the presence of the wild-type allele. This is in support of the protein measurements, as it was shown that variant alleles at this position are associated with higher FCN-2 concentrations [33]. As differences in allele frequencies in the analyzed *FCN2* polymorphisms were mostly lacking (with the exception of the association of the + 6359 polymorphism with FVC and diffusion capacity), these data may at first sight be consistent with an increase in FCN-2 concentrations (increased hepatic synthesis) as a consequence of an increased SSc activity or the progression of SSc over time. Longitudinal comparisons with FCN-2 concentrations in SSc patients before the disease onset are however lacking. We also can formally not exclude the possibility that high FCN-2 concentrations may induce Scl-70 autoantibody positivity or other mechanisms that trigger more severe SSc.

Our results are in line with a previous study of 90 SSc patients, which reported an association of higher FCN-2 levels with the presence of ILD and a reduced FVC [21]. In patients with idiopathic pulmonary fibrosis, above threshold levels of plasma FCN-2 were even associated with prolonged progression-free survival [34]. Whereas low FCN2 levels may predispose patients to pulmonary infections and bronchiectasis [35], increased FCN2 levels as observed in patients with ILD in the present study may trigger or maintain a detrimental inflammatory response to stressed endothelial cells in the lung [36] ultimately resulting in the development of clinically relevant ILD. Indeed, FCN-2 was also shown to participate in the clearance of dying cells maintaining tissue homeostatis [37] and hence may influence the induction of autoimmunity through mechanisms of the innate immune system [38]. Although complement deposition has been detected in pulmonary, renal, and dermal vessels [39–41], mechanistic data regarding local FCN-2 concentrations in the lung and their effect on endothelial and vascular injury and fibroblast activation are however currently lacking.

Our results complement previous studies with regards to MBL and FCN-3, which are two other important PRRs of the lectin pathway [21, 22]. In a study of 90 Australian SSc patients, higher MBL levels were significantly associated with vascular dysfunction, local tissue damage, skin involvement, and ILD [21]. With the exception of an association of higher MBL concentrations with scleroderma renal crisis, our study does not support a significant involvement of MBL in the pathogenesis of SSc, at least not when measured in the serum or when assessed by genotyping. Although local complement activation has been described in scleroderma renal crisis [42, 43], the involvement of the lectin pathway as compared to the classical or alternative pathway has never been investigated previously. Thus, our results warrant confirmation in a larger set of patients with scleroderma renal crisis.

Differences in cohort characteristics may account for some of the observed discrepancies, as the present study predominately included Caucasians (97% vs. 84% vs. 0% in previous studies [21, 22]) and a significant proportion of sine scleroderma SSc patients (15% vs. 1% vs. 0% in previous studies [21, 22]). SSc is a heterogeneous disease, and hence, analyses of the lectin pathway in subgroups based on clinical phenotype or the presence of certain autoantibodies may be desirable but requires a larger study.

FCN-3 serum concentrations were not associated with any of the investigated parameters or outcomes in the present study. Miyagawa et al. had however previously reported significantly lower FCN-3 serum concentrations in diffuse vs. limited cutaneous SSc patients, and a trend towards a higher prevalence of ILD in SSc patients with lower FCN-3 concentrations [20]. In line with the function of FCN-2, clearance of apoptotic cells is also mediated by FCN-3 [44]. Given that FCN-3 is also produced in the lungs, sufficient pulmonary FCN-3 levels may prevent extensive local inflammation as a consequence of apoptosis, and hence, lower levels may theoretically predispose SSc patients to interstitial lung disease. However, data regarding pulmonary FCN-3 levels are lacking and the small cohort size (*n* = 48) of the previous study and differences in ethnic background (100% Asian vs. 97% Caucasian in the present study) limit the significance of the observed FCN-3 results.

While the present study provides evidence for the involvement of circulating FCN-2 in SSc patients, our results do not exclude a participation of other lectin pathway proteins in the local regulation of complement activation in SSc [45]. Indeed, the evidence regarding the involvement of the lectin pathway in acute IR injury is convincing in rodents [46, 47] and even in humans [15, 48], although its importance in the setting of chronic low-grade ischemia, atherosclerosis, and vascular damage is less well defined [30, 49]. For example, whereas high-producing *MBL2* genotypes were associated with an increased risk of future ischemic heart disease and myocardial infarction in patients with rheumatoid arthritis [49], the opposite was true with respect to the development of myocardial infarction in diabetic and hypercholesterolemic individuals [50]. In addition, different pathophysiological mechanisms dominate over time in SSc, and the complement system may play a differential role depending on the disease duration. For example, microvascular dysfunction is a hallmark of early SSc, and the lectin pathway may contribute to inflammation during this stage. In contrast, the complement system may have limited impact at the fibrotic stage of SSc. Analyses of the lectin pathway during different disease stages are required to verify this hypothesis.

Limitations of the present study include the post hoc analysis of a predefined number of PRRs of the lectin pathway and the multiple statistical comparisons investigated. In addition, the sample size may curb the detection of small differences of lectin pathway proteins or polymorphisms, especially when analyzing rare events such as scleroderma renal crisis. The analysis of predominately Caucasian patients (97%) and of only a small percentage of patients with diffuse SSc (18%) may limit the generalizability of our study results.

## Conclusion

In conclusion, the present study results do not indicate a relevant role for several lectin pathway complement proteins in the pathogenesis of SSc. However, our observation of higher FCN-2 levels in SSc patients with digital vasculopathy and ILD is notable and requires validation.

## Abbreviations

FCN: Ficolin
IR: Ischemia/reperfusion
ILD: Interstitial lung disease
IQR: Interquartile range
MASP: Mannose-binding lectin-associated serine-protease
MBL: Mannose-binding lectin
PRR: Pattern recognition receptor
SD: Standard deviation
SSc: Systemic sclerosis
